# Transmission of human African trypanosomiasis in the Komo-Mondah focus, Gabon

**DOI:** 10.4314/pamj.v8i1.71151

**Published:** 2011-04-01

**Authors:** Tongué Lisette Kohagne, Mengue Paulette M’eyi, Raceline Gounoue Kamkuimo, Dramane Kaba, J Francis Louis, Rémy Mimpfoundi

**Affiliations:** 1 Organisation de Coordination pour la lutte contre les endémies en Afrique centrale (OCEAC), BP 15665, Yaoundé, Cameroun; 2 Programme national de lute contre la trypanosomiase humaine du Gabon, BP 940, Libreville, Gabon; 3 Laboratoire de Biologie Générale, Faculté des Sciences, Université de Yaoundé I, BP 812, Yaoundé, Cameroun; 4 Institut Pierre Richet/Institut national de Santé Publique, Abidjan, Côte d’Ivoire

**Keywords:** Epidemiology, entomology, transmission, trypanosomiasis, Glossina, Gabon

## Abstract

**Background:**

Knowledge about transmission of sleeping sickness in a given focus is of a great importance since it governs the efficacy and the cost-effectiveness of control strategy. The Komo-Mondah focus is the most endemic sleeping sickness focus of Gabon. This focus has hardly been investigated and available publications are more than thirty years old. In order to update transmission features of sleeping sickness in that focus, we have conducted epidemiological and entomological surveys in March-April 2008.

**Methods:**

Epidemiological investigation relied on a case-control study using a quantitative and qualitative methodology (a structured questionnaire). Cases were affected people (parasitological positive) diagnosed by the national control program from 2004 to 2007, controls were those found disease-free after clinical examination and biological tests in the same period. They were asked to respond to a standard questionnaire concerning their activities after having signed a written consent. An unvaried analysis was first performed and then a multivariate analysis using the conditional logistic regression for matching method. Traps were then set out for four days in areas where people were working. Tsetse flies captured were identified and dissected; their density and human-fly contact points were determined.

**Results:**

A risk of infection was associated with fishing activities (Odds-ratio: 5.69; CI95%: 3.38-9.57). Three species of Glossina were captured: Glossina palpalis palpalis, Glossina fuscipes fuscipes and G. Caliginea. Human-fly contact points were mainly landing stages.

**Conclusion:**

A combined strategy of case-detection and vector control targeted at landing stages should be efficient against the disease.

## Background

The epidemiology of human African trypanosomiasis (HAT) also call sleeping sickness due to *Trypanosoma brucei gambiense* has, for a long time, been thought to be determined essentially by human-fly contact and infection rates in the tsetse fly vector [1]. Rising and/or higher incidence of HAT in a given community relative to others have been linked to more intense human-fly contact, which can be a consequence of several factors: a higher density of the tsetse fly population, a spatial distribution which brings the flies closer to humans due to ecological changes, absence of alternative animal food sources resulting in tsetse taking a higher proportion of blood meals in humans, or humans spending more time in areas with denser tsetse populations [2]. Humans are exposed to the disease as far as it is in the vicinity of tsetse-infested areas. Various human activities such as fishing, farming, hunting, herding or water-related activities are known to be risk factors for acquiring sleeping sickness [3]. However, in some foci, the transmission is peri-domestic and humans do not need to be in a specific area of the focus to contract the disease [4]. Knowledge about transmission of sleeping sickness in a given focus is of a great importance since it governs the efficacy and the cost-effectiveness of control strategy.

The Komo-Mondah focus is the most endemic sleeping sickness focus of Gabon. Since 2003, populations of that focus are regularly examined by the national sleeping sickness control program (“Programme National de Lutte contre la Trypanosomiase Humaine Africaine” - PNLTHA), and more than thirty new cases are actively detected per year (PNLTHA, personal communication); sleeping sickness cases found in that focus correspond to 65% of the total number of new cases diagnosed in the whole country.

In spite of regular mass screening surveys performed in that focus, the incidence of the disease remains considerable; suggesting the need to put in place another control strategy. However, that focus has been less investigated and modalities of transmission are less known. As far as we are aware, only long-standing publications that date back to more than thirty years are available [5,6]. These authors pointed out the presence of Glossina around houses built at riverside, along footpaths, in farms, in fishing jetties or landing stages and in timber yards with fishermen and lumberjacks or woodcutters being more exposed than others.

Epidemiology of sleeping sickness is linked to human behaviour that could change in the course of time. In order to update knowledge about transmission of sleeping sickness in the Komo-Mondah focus, we carried out epidemiological and entomological surveys with the aim to put in place a suitable strategy in the fight against the disease.

## Methods

**Geographic zone**

The Komo-Mondah focus is located less than 50 km from Libreville (capital of Gabon). It extends over several villages from Ntoum (chief town of Komo-Mondah department) to Cocobeach (chief town of Noya department). It is located in the coastal area of the country and is adjacent to an active HAT focus of Equatorial Guinea: the Kogo focus. Around thirteen villages are located in that area but the disease concerns only six villages: Akok (00°51’N, 009°74’E), Ngouandji (00°58’N, 009°70’E), Nô Ayong (00°62N, 009°68’E), Biyemame (00°65’N, 009°66’E), Milembié (00°67’N, 009°64’E) and Bissobinam (00°95’N, 009°58’E) (fig.1). The climate is equatorial type characterised by important precipitation up to 2500 mm in rainy seasons, with temperatures varying from 23° to 32°C [7]. There are two types of vegetations, namely: mangrove at the maritime coastline and forest between the sea and villages [8]. In this forest, different ecological features overlap: foods crops, fallow land, and islands of secondary forest. Cocoa and coffee plantations are absent. The area is well irrigated by rivers that run into the Atlantic Ocean.

**Populations**

The population of Komo Mondah focus is ethnically homogeneous and speaks mainly Fang. The majority of the population is settled in Libreville for their activities (study or civil service) and villages are populated only during weekends and school holidays. The predominant activities in the area are fishing, farming (food crops), hunting, civil servants (teachers for primary school, nurses), grocers and woodcutters. A sizeable foreign population exists in the area (from Senegal, Togo, Cameroon, and Equatorial-Guinea, etc…). Domestic animals are rare in the area. Few sheep, goat, poultry, cats and dogs can be found around houses.

**Epidemiological survey**

This survey used a case-control design and was conducted from March to April 2008, during schools holidays when villages are populated. An administrative authorization was obtained from the Ministry of Health of Gabon to conduct the study. We didn’t conduct a mass screening survey, patients included in the study were those previously diagnosed by the national control program from 2004 to 2007. Thus, we firstly reviewed records of the national control program; relevant demographic data (name, village of origin, age, gender and the year of the diagnosis) of HAT patients diagnosed during the concerned period were collected, only patient positive for HAT were included in the study. Patients aged less than 10 years old were excluded from the line listing. Data were also collected from individuals registered and declared HAT-free during the same period. HA-free status was based on clinical examination, serological (CATT-Card Agglutination Test for Trypanosomiasis) and parasitological negatives tests.

All patients (positive and negative) identified from the list were tracked with the help of the village head (or chief) and village nurse. Each HAT patient found (some of them were dead or had travelled) was paired with two HAT-free controls randomly found, of the same age (0-5 years difference) sex and village, either living in the same or in a neighbouring village. Cases and controls were interviewed using a structure questionnaire. Patients were fully informed on the objectives of the study and were requested to provide their informed consent.

**Questionnaire**

For each case and their controls, data related to socio-demographic status, activities while being in the focus: fishing, farming, hunting, forestry activity; domestic activities in relation with water such as bathing, washing clothes; source of water for household use; recent history of travel (displacement) inside the focus were collected using a structure questionnaire. Quantitative and qualitative data related to risk of exposures, premises, period of the year, frequency and duration, the presence and the type of water spot were also collected.

**Entomological survey**

Working places were prospected and four biotopes were retained for trapping: border of the village, water spots, farms (food crops) and landing stages. “Vavoua” traps [9] were then set out in these biotopes of each village. They were cleared to ensure reasonable visibility and were examined twice a day, at 10 a.m. and at 4 p.m., during four days. Data on the species and sex of tsetse captured in each trap were recorded according to the computer-based identification key for tsetse flies [10]. Flies were dissected 24h after capture in a drop of sterile 0.9% saline water on a microscope slide under a magnifying glass. Midgut, proboscis and salivary glands were examined under a light microscope at a 400x magnification for the search of trypanosomes. Teneral flies were identified by the residual sac from the larval stage in the midgut [11]. Blood meals from flies were collected on Whatman number 4 papers and stored with desiccant in dark and dry conditions until analysis by ELISA for searching of their origin (human or animal).

**Treatment and data analysis**

Data were analysed using the computer package SPSS for Windows version 12.0. A univariate analysis was first performed, then a multivariate analysis using the conditional logistic regression for matching method [12]. Geographic distribution of HAT cases was analysed using the aggregation coefficient k (binominal parameter) [13,14] as follows: *k*=*X^2^/S^2^ - X* (X^2^ = mean, S^2^=variance). The smaller the k value, the greater the aggregation; k value greater than 8 indicates a random aggregation.

The human-fly contact index was calculated as follows: *p=(k × n × C^a-1^)/Pj^a^* [15]; with k a constant equal to 632 in farms and 623 at the edge of villages, n the number of human blood meals, C the number of captured flies; a is a constant equal to 1.23 in farms and 0.63 at the edge of villages, P the number of used traps, j the number of trapping days and t the number of teneral flies.

## Results

A total of 133 HAT positive patients were registered, 120 patients could be located found but only 106 were included in the study. Case-patients were distributed by village as follows: Akok (6), Ngouandji (32), Nô Ayong (18), Biyemame (18), Milembié (12), Bissobinam (20).

**Socio-demographic characteristics**

Out of 318 participants included in the study (106 cases and 212 controls), 64.15% were men and 35.85% were women (men/women sex-ratio = 1.78). Socio-demographic characteristics of case are compared to controls in table 1. Pairing criteria retained for the study were totally respected.

**Risk exposure and spatial aggregation**

The study links fishing to the disease (table 2): thus, fishers are at risk to contract sleeping sickness, estimated by the odds ratio, more than five times than others (OR = 5.69; CI 3.38-9.57, p

An average of more than one HAT case were found per house in each village as follows; Akok (1.40 ±0.55), Ngouandji (2.21±0.97), Nô Ayong (1.8±0.79), Biyemame (1.50±0.52), Milembié (1.50±0.53) Bissobinam (1.42±0.65). There was a spatial aggregation of HAT cases in all villages screened; the coefficient k varied from 1.45 to 3.87 in screened villages.

**Distribution of the vector and human-fly contact points**

A total of 1251 tsetse flies were captured in the six villages; Glossina were present at the border of only one village. They were present in water spots and landing stages of all screened villages and in farms in some villages. Apparent density of tsetse per trap (ADT) was significantly different per biotopes and per villages (X^2^=12.14; P*Glossina palpalis palpalis* (1149; 91.85%), *Glossina fuscipes fuscipes* Newstead, 1911 (85; 6.79%) and Glossina caliginea Austen, 1929 (17; 1.36%). *G. palpalis palpalis* was found in all biotopes while *G. Caliginea* and *G. fuscipes fuscipes* were found only in water spots and landing stages.

Teneral flies of *G. palpalis palpalis* were identified in water spots and landing stages of all villages; those of *G. fuscipes fuscipes* were found only in landing stages of one village. No *G. Caliginea* teneral fly was captured. Human-fly contact was identified in landing stages of all screened villages, in water spots of two villages and in farms of only one village.

## Discussion

Transmission of *Trypanosoma brucei gambiense* sleeping sickness is enhanced by increased contact between *Glossina* and humans [16]. We observed that fishing is currently the main activity that exposed people of this focus to the disease, contrary of earlier investigations [5,6] which demonstrated a link between fishing, forestry activity (woodcut) and the disease. Human’s activities determine the transmission of HAT since they led people into tsetse infested areas.

In years that follow the independence of the country, many timber yards was implemented in Gabonese forest. This activity brought populations in contact with *Glossina* which was abundant in the area thanks to suitable environmental conditions provided by many rivers that crossed the focus. Besides, villagers were for the most part in camps built along rivers. Nowadays, forestry activity has declined considerably. The geographical position of the focus (between two townships: Ntoum and Cocobeach) has brought “modernization” in the area. Tsetse flies, influenced mainly by density-independent factors such as temperature and humidity [17] are mainly confined in water spots, landing stages and also in farms in some villages, due to the destruction of the vegetation cover. *Glossina* is absent around houses built along the main road axe; except in one village. Humans need to visit these infested biotopes to contract the disease. This occurs particularly when they are boarding or landing before or after fishing at sea, landing stages being the most infested biotopes. Almost half of HAT patients belongs to the active part of the population, because of their main activity (fishing) while being in the area. This was also observed in mangrove swamp of Guinea [18] and in Côte d’Ivoire forest [19].

Fishing is run by natives, especially men and this could explain the fact that men are more exposed to the disease than women. Students concerned by the disease are those accompany their parents for fishing. Natives are numerous and therefore more concerned by the disease than foreigners. Nevertheless, one HAT case originated from Equatorial Guinea was also identified. This patient was a fisherman and at the end of our epidemiological survey, we couldn’t determine exactly where he has been infested. This suggests that, fishermen who travel easily from a focus (Gabon) to another one (Equatorial Guinea) in the course of their activities, contribute to the spread of the disease.

The low value of the aggregation coefficient k induces a familial concentration that has been considered as an important epidemiological property of sleeping sickness in the central Africa region [20,21]. Considering that *Glossina* are rare around houses and human needs to be in a particular area to be infested, we could argue that this familiar concentration is a consequence of, either the fact that members of the same family are sharing the same biotope and are similarly (not simultaneous) exposed to tsetse, or to a simultaneous exposure to an infective tse-tse whose blood meal from a first individual was interrupted and resumed minutes later on a second and possibly third nearby relative, infecting all of them [2].

According to the National Sleeping Sickness Control Program, Bissobinam is the most endemic village in Gabon (PNLTHA, unpublished). Our results however do not confirm that observation as HAT cases were mainly reported in Ngouandji. Nevertheless, Bissobinam was the only village where tsetse flies were caught in all biotopes investigated, even at the border of houses. This village is more irrigated than others with rivers close to the houses. *Glossina* of the *palpalis* group are known to be close to water, where relative humidity (up to 65%) could prevent them from dessication [21]. The location of rivers nearby that village could explain the omnipresence of tsetse flies in the village. This hypothesis is also valid for villages where tsetse flies were caught in farms. Species of tse-tse flies captured were similar to those observed during earlier investigations [5, 6,8] and their distribution has not really changed. G. *palpalis palpalis* was found in all biotopes while G. *fuscipes fuscipes* were found only in water spots and landing stages in Akok. These two species are known to compete while being in the same area, with G. *Palpalis* pushing away *G. Fuscipes* [22]. It is difficult for us to assert such a thing although; they were not captured in the same biotope.

Transmission of *T. b. Gambiense* trypanosomiasis is not determined by the density of vector but rather by the closeness of human-fly contact [23]. Landing stages were identified as human-fly contact points in all villages. The presence of water in an endemic area increases the risk for contracting the disease by more than three folds [23].

## Conclusion

Transmission of sleeping sickness in the Komo-Mondah focus is particularly linked to fishing. The risk to contract the disease is not really associated to the activity, but rather to the frequence of tsetse infested biotopes such as landing stages and some water spots. National HAT control program should put in place a control strategy involving both case-detection and vector control activities, targeted at human-fly contact points.

## Acknowledgements

We thank administrative and sanitary authorities of Komo-Mondah and Noya departments. We are grateful to the populations of Komo-Mondah focus for their collaboration. Technical assistance was provided by the technical staff of PNLTHA/Gabon and PSR-THA/OCEAC. We are grateful to Dr Nkinin and Dr Ndassa for the very useful criticisms of the manuscript. This research work was financed by OCEAC within the framework of support to countries members.

## Competing interests

Authors declared no competing interests.

## Authors’ contributions

LKT: Conception and design, technical task, interpretation of data, drafting the paper. PMM: Technical task. DK: interpretation of data, manuscript revision. RGK: Technical task, manuscript revision. FJL: Conception and design, manuscript revision..RM: manuscript revision. All the authors have read and approve the final version of the manuscript

## Figures and Tables

**Table 1:** Socio-demographic characteristics cases and controls

Demographic variables	HAT positive	Controls
	(n = 212)	(n = 106)
Mean age (to ET)	37.2 ± 14.2	37.2 ± 14.2
**Sex**		
Female	38 (35.85%)	76 (35.85%)
Male	68 (64.15%)	136 (64.15%)
**Age groups**		
< 20 years old	16 (15.09%)	32 (15.09%)
20-49 years old	75 (70.76%)	150 (70.76%)
>49 years old	15 (14.15%)	30 (14.15%)

HAT, human African trypanosomiasis; ET=σ, standard deviation

**Table 2:** Human activities and association with sleeping sickness in the Komo- Mondah focus

	HAT patients	Controls		95% confidence	
	n = 106 (%)	n = 212 (%)	OR*	intervals	P value
Fishing	79 (74.53)	72 33.96)	5.69	3.38-9.57	P<0.001
Studying	21 (19.81)	87 41.04)	0.35	0.20-0.60	P>0,90
Civil service	2 (1.89)	9 (4.25)	0.43	0.09-2.02	P> 0,30
Housework	4 (3.77)	13 6.13)	0.60	0.19-1.88	P>0,50
Forestry activity	0	14 6.60)	-	-	-
Grocery	0	11 (5.19)	-	-	-
Hunting	0	6 (2.83)	-	-	-

HAT: Human African Trypanosomiasis;

* Odd ratio: Estimation of maximum likelihood for matched Odds Ratio

## References

[1] Scott D, Mulligan HW, Potts WH The epidemiology of Gambian sleeping sickness.

[2] Khonde N, Pépin J, Niyonsenga T, De Wals P (1997). Familial aggregation of Trypanosoma brucei gambiense trypanosomiasis in a very high incidence community in Zaïre. Trans R Soc Trop Med Hyg.

[3] Laveissière C, Hervouet JP, Couret D (1986). Localisation et fréquence du contact homme-glossine en secteur forestier de Côte d’Ivoire: 2 Le facteur humain et la transmission de la trypanosomiase. Cah ORSTOM Sér Ent Parasitol.

[4] Gouteux JP, Kounda Gboumbi JC, Noutoua L, D’Amico F, Bailly C, Roungou JB (1993). Man-fly contact in the Gambian trypanosomiasis focus of Nola-Bilolo (Central African Republic). Tropical Medicine and Parasitology.

[5] Challier A Enquête sur les glossines des foyers de trypanosomiase humaine en République du Gabon - Prospection des gîtes de l’Estuaire et de l’Ogooué Maritime. Recommandations pour une campagne de lutte. Rapport final Organisation mondiale de la santé.

[6] Mboumba M (1977). Lutte contre les glossines au Gabon, foyer de l’Estuaire. Rapport final du Service National d’Assainissement.

[7] Sarda J (1985). La trypanosomiase au Gabon en 1985. Bull Liais Doc OCEAC.

[8] Lancien J, Sarda J, Ekouague L, Bekale (1986). Une première expérience de lutte par piégeage contre les mouches tsé-tsé dans la mangrove gabonaise. Cah ORSTOM Sér Ent Méd Parasito.

[9] Laveissière C, Grébaut P (1990). Recherches sur les pièges à glossines (Diptera: Glossinidae) - Mise au point d’un modèle économique: le piège Vavoua. Trop Med Parasitol.

[10] Brunhes J, Cuisance D, Geoffroy B, Hervy JP, ORSTOM (1998). Les glossines ou mouches tsé-tsé. Logiciel d’identification et d’enseignement.

[11] Laveissière C (1975). Détermination de l’âge des glossines ténérales. Cah ORSTOM sér Ent Méd Parasitol.

[12] Dallas GE (1989). CLOGISTIC: a conditional regression program for the IBM-PC. The Am Stat.

[13] Southwood TRE (1968). Ecological methods.

[14] Gouteux JP, Noireau F, Malongo JR, Frézil JL (1988). "Effet de case" et "contamination familiale" dans la maladie du sommeil; essai d’interprétation du phénomène - Exemple de trois foyers congolais. Ann Parasitol Hum Comp.

[15] Laveissière C, Sané B, Méda HA (1994). Measurement of risk in endemic areas of human african trypanosomiasis in Côte d’Ivoire. Trans R Soc Trop Med Hyg.

[16] Leak SGA Tsetse biology and ecology. Their role in the epidemiology and control of trypanosomiasis.

[17] Baldry DAT (1980). Local distribution and ecology of Glossina palpalis and Glossina tachinoides in forest foci of west African human trypanosomiasis, with special reference to associations between peri-domestic tsetse and their hosts. Ins Sci Appl.

[18] Camara M, Kaba D, Kagbadouno M, Sanon JR, Kakila E, Solano P (2005). La Trypanosomose Humaine Africaine en zone de mangrove en Guinée: étude épidémiologique et clinique de deux foyers voisins. Med Trop.

[19] Laveissiere C, Hervouët JP, ORSTOM Trypanosomiase Humaine en Afrique de l’Ouest Epidémiologie et contrôle.

[20] Frézil JL, Eouzan JP, Alary JC, Malongo JR, Ginoux AY (1980). Epidémiologie de la trypanosomiase humaine africaine en République Populaire du Congo - II - Le foyer du Niari. Cah ORSTOM Sér Ent Parasitol.

[21] Nash TAM (1937). Climate: the vital factor in the ecology of Glossina. Bull Entomol Res.

[22] Gouteux JP (1992). Un cas d’exclusion géographique chez les glossines: l’avancée de G palpalis palpalis vers Brazzaville (Congo) au détriment de G palpalis quanzensis. Insect Sci Applic.

[23] Laveissière C, Hervouet JP, Couret D (1986). Localisation et fréquence du contact homme-glossine en secteur forestier de Côte d’Ivoire - 1 Recherche des points épistémologiquement dangereux dans l’environnement végétal. Cah ORSTOM Sér Ent Parasitol.

